# Extracellular Monomeric Tau Protein Is Sufficient to Initiate the Spread of Tau Protein Pathology*

**DOI:** 10.1074/jbc.M113.515445

**Published:** 2013-11-14

**Authors:** Claire H. Michel, Satish Kumar, Dorothea Pinotsi, Alan Tunnacliffe, Peter St. George-Hyslop, Eckhard Mandelkow, Eva-Maria Mandelkow, Clemens F. Kaminski, Gabriele S. Kaminski Schierle

**Affiliations:** From the ‡ Department of Chemical Engineering and Biotechnology, University of Cambridge, Pembroke Street, Cambridge CB2 3RA, United Kingdom,; the § German Center for Neurodegenerative Diseases (DZNE), Ludwig-Erhard-Allee 2, 53175 Bonn, Germany,; the ¶ MPI for Neurological Research, Hamburg Outstation, c/o Deutsches Elektronen-Synchrotron, Notkestrasse 85, 22607 Hamburg, Germany,; the ‖ Cambridge Institute for Medical Research, Wellcome Trust/MRC Building, Addenbrooke's Hospital, Hills Road, Cambridge CB2 0XY, United Kingdom,; the ** Tanz Centre for Research in Neurodegenerative Diseases, University of Toronto, Ontario, M5S 3H2, Canada, and; the ‡‡ Center of Advanced European Studies and Research, Ludwig-Erhard-Allee 2, 53175 Bonn, Germany

**Keywords:** Alzheimer Disease, Amyloid, Endocytosis, Protein Aggregation, Tau, Fluorescence Lifetime Imaging Microscopy, Propagation, Superresolution Microscopy

## Abstract

Understanding the formation and propagation of aggregates of the Alzheimer disease-associated Tau protein *in vivo* is vital for the development of therapeutics for this devastating disorder. Using our recently developed live-cell aggregation sensor in neuron-like cells, we demonstrate that different variants of exogenous monomeric Tau, namely full-length Tau (hTau40) and the Tau-derived construct K18 comprising the repeat domain, initially accumulate in endosomal compartments, where they form fibrillar seeds that subsequently induce the aggregation of endogenous Tau. Using superresolution imaging, we confirm that fibrils consisting of endogenous and exogenous Tau are released from cells and demonstrate their potential to spread Tau pathology. Our data indicate a greater pathological risk and potential toxicity than hitherto suspected for extracellular soluble Tau.

## Teaser

**Background:** The aggregation and stereotypic spreading of Tau protein is associated with Alzheimer disease.

**Results:** Monomeric Tau enters neurons and nucleates and engages endogenous Tau to aggregate.

**Conclusion:** Endocytosis of soluble Tau triggers aggregation in vesicles and is sufficient to initiate the spreading of pathological species.

**Significance:** Increased levels of extracellular monomeric Tau may increase the risk of developing tauopathies.

## Introduction

Accumulation of misfolded proteins is a specific feature of neurodegenerative diseases. Until recently it was thought that protein misfolding in Alzheimer disease (AD)^3^ and related neurodegenerative diseases is a cell-autonomous process, in contrast to prion diseases, where pathological aggregates can spread from cell to cell. In recent reports, however, a prion-like spreading of neurotoxic aggregates has also been suggested for non-prion-related neurodegenerative diseases (see Refs. 1–5). These efforts have partly been driven by the fact that, in AD patients, the neurofibrillary pathology of Tau has been observed to spread in a well defined pattern that correlates with the clinical stages of the disease (Braak stages 1–6 (6)). This is in contrast with the pattern observed for Aβ deposits in AD, which follows a less defined path (7). The molecular basis of the cell-to-cell spreading of Tau pathology has been a matter of intense debate. Mechanisms have been suggested that relate Tau to Aβ (8), inflammation (9), and others. The hypothesis that Tau itself might contribute to the propagation of the disease is more recent, and the latest studies using animal or cellular models of AD strengthen the notion of a prion-like propagation of Tau pathology between cells (10–18).

Although the above findings have provided evidence that Tau can traffic between cells in different model systems, it is still uncertain how Tau aggregation proceeds in these models. Also, the methods used so far have not allowed for a continuous monitoring of the aggregation state in live cells, yet such information is crucial for an understanding of the molecular pathology of the disease and in the exploration of therapeutic interventions.

To address these issues, we took advantage of recent developments in our laboratories that enable the monitoring of aggregate-forming reactions via fluorescently labeled proteins. In particular, we exploit changes in the fluorescence lifetime of reporter fluorophores to track the dynamics of aggregation with high sensitivity. We have shown previously that the fluorescence lifetime decreases in proportion with the appearance of β sheet-containing structures (19–21) and, thus, offers structural information on the aggregation state that cannot be obtained by intensity-based imaging. Our approach requires only single fluorophore species for labeling and is independent of label concentration, an advantage over two-color FRET methods reported previously (15). There are two consequences: 1) it becomes possible to monitor the coaggregation of labeled and non-labeled species, crucial in a study of the interaction of internalized and endogenous protein species; and 2) using a low dye labeling ratio on the peptide scaffold minimizes the potential for steric interference with the aggregation process. These advantages are essential for live-cell studies of amyloid translocation.

In this work, we systematically analyze cellular uptake, vesicular trafficking, and the transfer of initially monomeric Tau between cells by extending the above described methodology. Furthermore, two-color *direct* stochastic optical reconstruction microscopy (*d*STORM) reveals that coaggregation of imported exogenous and endogenous Tau takes place and that the resulting aggregates can readily infect healthy cells. In summary, our data provide strong evidence that endocytosis of monomeric Tau is sufficient to initiate Tau pathology.

## EXPERIMENTAL PROCEDURES

### Protein Preparation

Full-length human Tau (hTau40) and its four-repeat construct (K18) were prepared as described previously (22). For this study, we replaced the native cysteines at 291 and 322 of both hTau40 and K18 constructs with alanines and replaced Ile-260 with a cysteine for labeling with Alexa Fluor 488 and 647 (these triple mutants are termed hTau40* and K18*). This approach allows labeling at the defined residue at 260 and avoids the presence of a bulky fluorescent dye within the core of the β-promoting region in R2 and R3 that would interfere with aggregation.

### Labeling of Proteins

Proteins hTau40* or K18* were incubated in 1× BRB80 buffer (80 mm PIPES, 1 mm MgCl_2_, and 1 mm EGTA (pH 6.8)) with a 10-fold molar excess of tris-(2-carboxyethyl)phosphine at room temperature for 30 min for complete reduction of intermolecular disulfide bonds. Thereafter, a 4-fold molar excess of Alexa Fluor 488 maleimide (Life Technologies) dissolved in dimethyl sulfoxide was added to the protein solution, and labeling was allowed to proceed at room temperature for 3 h in the dark. The unlabeled fluorophores were separated from the labeled protein solution using a NAP-5 column (GE Healthcare) equilibrated previously with 1× BRB80 buffer. The protein concentration was determined by the BCA method and further confirmed by SDS-PAGE with subsequent Coomassie staining. The concentration of bound dye was determined by the molar extinction coefficient of Alexa Fluor 488 (ϵ495 = 72,000 cm^−1^ m^−1^). Typically, the labeling efficiency was 80–90%. The fluorescently labeled Tau protein was then flash-frozen and stored at −80 °C until use.

### Assembly of Tau Paired Helical Filaments and Electron Microscopy

Aggregation of unlabeled or Alexa Fluor 488-labeled K18* was induced by incubating 10 μm protein in volumes of 200 μl at 37 °C in 50 mm NH_4_ acetate, 1 mm DTT (pH 7.0) containing 2.5 μm heparin (*M*_r_ 3000). Aggregation of unlabeled or Alexa Fluor 488-labeled hTau40* was done similarly, except in N,N-bis(2-hydroxyethyl)taurine (BES) buffer (20 mm BES with 25 mm NaCl and 1 mm DTT (pH 7.4)) containing 2.5 μm heparin (*M*_r_ 3000). Aggregated labeled and unlabeled Tau was visualized by negative stain electron microscopy. Protein solutions were placed on 600 mesh carbon-coated copper grids for 45 s, washed twice with H_2_O, and negatively stained with 2% uranyl acetate for 45 s. The samples were examined with a Philips CM12 electron microscope at 100 kV.

### In Vitro Tau Preparation for Fluorescence Lifetime Imaging Microscopy

The constructs were used either unlabeled or labeled with Alexa Fluor 488 as described above. *In vitro* Tau aggregation was induced by incubating the peptide with heparin at a molar ratio of 4:1 Tau:heparin (*M*_r_ 3000) for 24 h. Aggregation of K18* was always performed in cell culture medium, whereas aggregation of hTau40* was performed in BES buffer (20 mm BES and 25 mm NaCl (pH 7.4)) because of its low propensity to aggregate.

### Cell Culture

SH-SY5Y and pgsA-745 cells were obtained from LGC Standards (Teddington, UK). SH-SY5Y cells were maintained on 1:1 minimal essential medium (MEM) (Sigma):nutrient mixture F12 Ham (Sigma), 15% FBS (Life Technologies), 1% l-glutamine (Sigma), 1% MEM non-essential amino acids (Sigma), and 1% antibiotic-antimycotic (10,000 units of penicillin, 10,000 μg of streptomycin, 25 μg of amphotericin B; Life Technologies). PgsA-745 cells were maintained on nutrient mixture F12 Ham with 10% fetal bovine serum. All incubations with K18* and hTau40* were carried out in serum-free medium by replacing the FBS with 2% B27 complement (Life Technologies). Extracellular Tau was eliminated by a trypsin wash. Cells were incubated with 0.01% trypsin (Life Technologies) in MEM for 1 min, with 10% FBS in MEM for 5 min, and with MEM for 5 min. This washing protocol ensured the complete digestion of remaining extracellular Tau by trypsin, followed by deactivation of the trypsin.

### Western Blotting

After a trypsin wash (see above), cells were lysed in 50 μl of Triton lysis buffer (1% Triton X-100, 50 mm Tris, 150 mm NaCl, and protease inhibitors (Roche Diagnostics) (pH 7.6)). The samples were centrifuged at 14,000 × *g* for 30 min, and the supernatant was collected (Triton fraction). The pellet was washed with Triton lysis buffer and then solubilized in sarkosyl lysis buffer (1% sarkosyl, 50 mm Tris, 150 mm NaCl, and protease inhibitors (pH 7.6)). The samples were centrifuged at 14,000 × *g* for 30 min, and the supernatant was collected (sarkosyl fraction). The pellet was washed with sarkosyl lysis buffer and then solubilized in SDS lysis buffer (1% SDS, 50 mm Tris, 150 mm NaCl, and protease inhibitors (pH 7.6)) to form the SDS fraction. The different conditions were separated by gel electrophoresis on a NuPAGE® Novex 4–12% BisTris gel in NuPAGE® MES SDS running buffer (Life Technologies). The proteins were transferred onto a PVDF membrane (Millipore, Watford, UK). The membranes were incubated with 1/2000 polyclonal rabbit anti-human Tau antibody A0024 (DAKO, Glostrup, Denmark) and 1/5000 anti-rabbit IgG-peroxidase (Sigma). Proteins were revealed with SuperSignal West Pico chemiluminescent substrate (Thermo Fisher Scientific, Cramlington, UK) on a Syngene GBOX Chemi XT4 gel documentation system.

### Confocal Microscopy

#### 

##### Colocalization Study with FM 4-64

SH-SY5Y cells were incubated with 1 μm 10% K18*-488. FM® 4-64 (Life Technologies) was added 24 h after K18*-488 addition and incubated for 15 min before washing the cells with trypsin.

##### Uptake at 4 °C

SH-SY5Y cells were incubated with 1 μm 10% K18*-488 either at 37 °C (control) or at 4 °C for 1 h, followed by trypsin wash. Cells were observed on a Leica SP5 confocal microscope (Leica Microsystems GmbH, Wetzlar, Germany). Samples were imaged using a 488-nm excitation wavelength and a 500- to 530-nm emission filter to visualize K18*-488. To visualize FM^®^ 4-64, samples were imaged with a 543-nm excitation wavelength and a 700- to 800-nm emission filter.

### Fluorescence Lifetime Imaging Microscopy

*In vitro* samples were placed in silicon gaskets (Life Technologies) on a coverslip. Live cells were incubated in glass-bottom dishes (MatTek Corp.) in a chamber at 37 °C and 5% CO_2_ onto the microscope stage. *In vitro* and *in vivo* samples were imaged on a home-built confocal microscopy setup as described (19). An excitation wavelength of 488 nm was used, with a 525/39 bandpass emission filter. Images were acquired for 100–300 s, and photobleaching was verified to be negligible during these acquisition times. All TCSPC images were processed using SPCImage (Becker & Hickl GmbH, Berlin, Germany) and fitted with a monoexponential decay function. Image processing and data analysis were carried out with code developed in-house using Matlab (The Mathworks Ltd., Cambridge, UK).

### dSTORM Imaging

Cells were incubated with 1 μm K18* (either unlabeled or 10% labeled with Alexa Fluor 647) for 72 h, trypsin-washed, and incubated in Tau-free medium for a further 72 h before the medium was collected. The medium used to seed hTau40* was incubated for 48 h with 1 μm 10% hTau40*-647 in Lab-Tek II chambered coverglass (NUNC^TM^, Thermo Fisher Scientific) before being washed and imaged by *d*STORM. The medium analyzed by immunochemistry was incubated for 1 h in a Lab-Tek II chambered coverglass. The medium was then washed, blocked in 5% donkey serum in PBS, and probed with 1/200 TAUY9 rabbit polyclonal antibody (Enzo Life Science, Exeter, United Kingdom) and Alexa Fluor 568 secondary antibody (Life Technologies). For all *d*STORM imaging, quenching buffer was made from 400 μl glucose oxidase solution (0.02 mg/ml catalase, 4 mm tris-(2-carboxyethyl)phosphine, 50% glycerine, 25 mm KCl, 20 mm Tris-HCl, and 1 mg/ml glucose oxidase in water), 50 μl of glucose solution (100 mg/ml glucose and 10% glycerin in water), and 100 μl of 1 m mercaptoethylamine HCl (pH 7.4). This quenching buffer was added to the Lab-Tek chamber. The chamber was then filled to the top with PBS and sealed with a coverslip to avoid any entrance of oxygen. Single- and two-color superresolution imaging was performed on a home-built *d*STORM microscopy setup on the basis of a Nikon Eclipse TE 300 inverted wide-field microscope with a ×100, 1.49 numerical aperture total internal reflection fluorescence (TIRF) objective lens (Nikon UK Ltd., Kingston Upon Thames, UK) (23). For the two-color imaging, we used as excitation sources two laser lines at 640 nm (Toptica Photonics AG, Gräfelfing, Germany) (red channel) and 561 nm (Oxxius SLIM-561, Oxxius, Lannion, France) (green channel). They were collimated and combined by dichroic mirrors and a beam-expanding telescope. The laser beams were subsequently focused onto the back focal plane of the objective. A 405-nm (120-milliwatt) (Mitsubishi Electronics Corp., Tokyo, Japan) laser was used as reactivation source. To separate the individual emissions from the two channels (red and green), the fluorescence light in the detection path went through a dichroic filter (Semrock multi-edge filter Di01-R405/488/561/635-25 × 36 followed by a FF01–446/523/600/677-25 filter, Semrock, Rochester, NY) and was subsequently filtered further using band-pass filters (Semrock BP-607/35-25 and BP-642/35-25 for the green and red channels, respectively) before being projected on low-noise, highly sensitive electron-multiplying CCD camera (Ixon DV887 ECS-BV, Andor, Belfast, United Kingdom). To generate a final pixel size of 160 nm, additional lenses were placed in the detection path. The excitation intensity was 2 kW/cm^2^ for the Toptica laser and 5 kW/cm^2^ for the Oxxius laser. The reactivation laser was only turned on when the number of active fluorophores in the field of view was reduced. The two channels were imaged sequentially, the red channel followed by the green channel. No spatial drift of the sample was observed during the acquisition time of the two channels. Imaging was performed in TIRF illumination in all cases, at the center of an area consisting of 64 × 64 camera pixels, corresponding to a ∼10 × 10 μm^2^ area on the sample. In this case, any optical offset resulting in colocalization error was insignificant. Typically, 10,000 single-molecule frames with 10–12 ms of exposure time were recorded. The exposure time was matched with the “on” state of the fluorescent dyes. From each image stack, a reconstructed *d*STORM image was generated by using software developed in-house (24) on the basis of Matlab.

## RESULTS

### 

#### 

##### Labeling of Tau with Alexa Fluor 488 Does Not Interfere with Fibril Formation and Permits the Monitoring of the Aggregation State in Vitro

**FIGURE 1.**
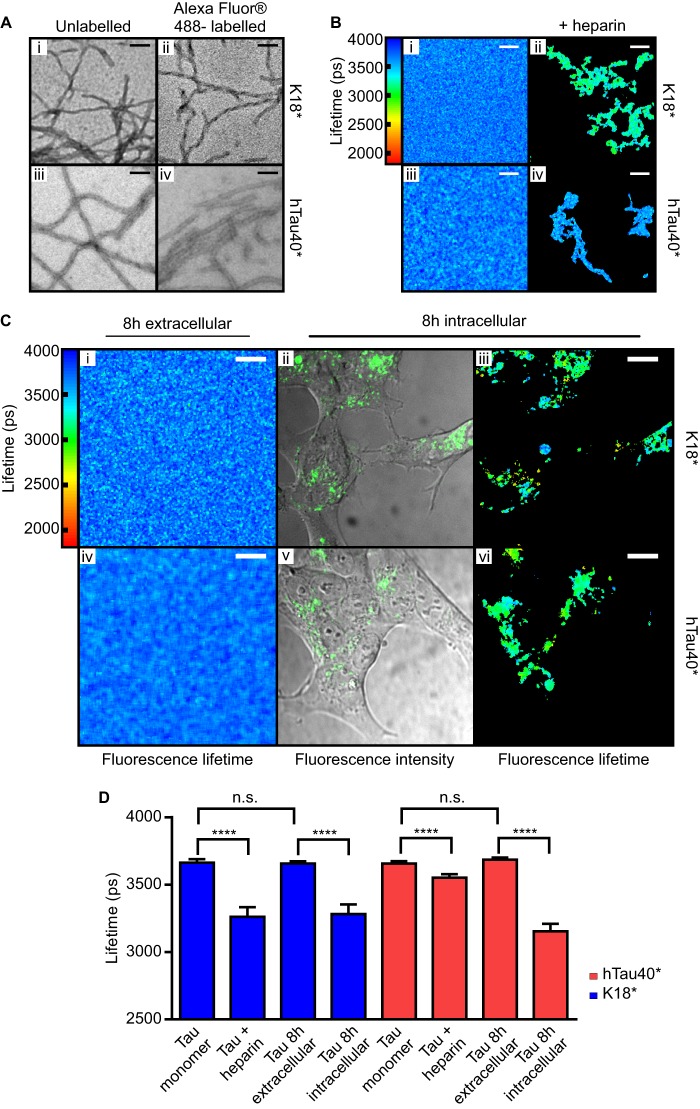
The fluorescence lifetime of Alexa Fluor 488-labeled K18* and hTau40* reports on the structural conformation of the protein *in vitro* and *in vivo*. *A*, K18* (*i*) and K18*-488 (*ii*), each incubated at 10 μm with 2.5 μm heparin for 6 h prior to transmission electron microscopy analysis, form similar fibrils. Similarly, hTau40* (*iii*) and hTau40*-488 (*iv*), each incubated at 10 μm with 2.5 μm heparin for 240 h prior to transmission electron microscopy analysis, form similar fibrils. *Scale bars* = 100 nm. *B*, the fluorescence lifetime of 10% K18*-488 (1 μm in cell culture medium) and 1% hTau40*-488 (20 μm in BES buffer) was either measured immediately (*i* and *iii* for K18* and hTau40*, respectively) or after incubation with 4:1 Tau:heparin for 24 h at 37 °C (*ii* and *iv* for K18* and hTau40*, respectively). Prior to inducing aggregate formation by heparin, the excited-state lifetime of K18*-488 lies at 3664 ± 25 ps (*i*) and that of hTau40*-488 at 3657 ± 18 ps (*iii*). After heparin treatment, the fluorescence lifetime of K18*-488 drops to 3262 ± 71 ps (*ii*), whereas that of hTau40*-488 drops to 3552 ± 26 ps (*iv*). *Scale bars* = 10 μm. *C*, 1 μm 10% K18*-488 or hTau40*-488 were added to the extracellular medium of SH-SY5Y cells, and their fluorescence lifetimes were analyzed after 8 h incubation (*i* and *iv* for K18* and hTau40*, respectively). The fluorescence lifetimes of the extracellularly added K18*-488 and hTau40*-488 remained at 3658 ± 16 ps and 3686 ± 16 ps, respectively, indicating that Tau does not aggregate in the absence of an aggregation-inducing agent, such as heparin, in the extracellular space. Confocal (*ii* and *v* for K18* and hTau40*, respectively) and TCSPC images (*iii* and *vi* for K18* and hTau40*, respectively) of SH-SY5Y cells, which were incubated for 8 h with 1 μm 10% K18*-488 or hTau*-488 and then trypsin-washed prior to imaging, are shown. The fluorescence lifetimes of K18*-488 and hTau40*-488 dropped to 3282 ± 72 ps and 3154 ± 56 ps, respectively. *Scale bars* = 10 μm. *D*, *bar diagram* displaying the mean fluorescence lifetime values of the different samples measured. *Error bars* represent S.D. One-way analysis of variance was performed for K18* (F (3, 102) = 616.7, *p* < 0.0001) and hTau40* (F (3, 74) = 980.5, *p* < 0.0001). ****, *p* < 0.0001; *n.s.*, not significant.

Using transmission electron microscopy, we verified that hTau40* and K18*, labeled with a single Alexa Fluor 488 moiety or unlabeled, form morphologically similar fibrils after incubation with heparin (Fig. 1*A*). Unless stated otherwise, we used mixtures containing 100 nm labeled Tau (*e.g.* K18*-488) and 900 nm unlabeled Tau (*e.g.* K18*) in all experiments to minimize potential steric interference of labels. We will subsequently refer to these mixtures as *e.g.* 1 μm 10% K18*-488.

Next, we verified that the fluorescence lifetime of Alexa Fluor 488 provides a read-out on the Tau aggregation state similar to that observed for YFP attached to a related protein, α-synuclein (19). We measured the fluorescence lifetime of K18*-488 and hTau40*-488 in either of their monomeric and aggregated states *in vitro* using a modified confocal microscope containing a TCSPC module (19, 25). We thus incubated 1 μm 10% K18*-488 for 24 h with heparin to trigger Tau aggregation (26) in culture medium. Fig. 1, *B* and *D*, shows that K18*-488 has a fluorescence lifetime of 3664 ± 25 ps in its soluble form (Fig. 1*B*, *i*, and *D*, *Tau monomer*) and 3262 ± 71 ps when aggregated (***B***, *ii*, and *D*, *Tau* + *heparin*). HTau40* is less aggregation-prone and was aggregated as 1% hTau40*-488 at 20 μm total protein concentration in BES buffer in the presence of heparin. The fluorescence lifetime of hTau40*-488 was 3657 ± 18 ps in its monomeric form and 3552 ± 26 ps when aggregated (Fig. 1*B*, *iii* and *iv*, and *D*, *Tau monomer* and *Tau* + *heparin*). These results are consistent with previous observations of strongly aggregated and less aggregated species (19) that have been observed for K18 and hTau40, respectively (27).

##### Monomeric Exogenous Tau Is Rapidly Endocytosed by SH-SY5Y Cells, and Tau Aggregation Is Favored by Low pH in Endosomes

*In vitro*, both K18* and hTau40* require the presence of agents such as heparin, sulfated glycosaminoglycans, or other polyanionic factors for their efficient aggregation (28). Because the cell membrane contains glycosaminoglycans, we asked whether the presence of cells affects the structural fate of monomeric Tau added to the medium. To this end, we added 1 μm 10% K18*-488 or 1 μm 10% hTau40*-488 to the growth medium of SH-SY5Y cells. TCSPC recordings were performed over 72 h but revealed no significant fluorescence lifetime changes either for K18*-488 or for hTau40*-488 remaining in the extracellular space (Figs. 1*C*, *i* and *iv*, and *D*, *Tau 8 h extracellular*, and 4*F*, *navy blue bars*). These data suggest that exogenous Tau is stable without the addition of heparin.

However, less than 1 h after the addition of Tau to the extracellular space, we observed its accumulation inside of SH-SY5Y cells, suggestive of an uptake of monomeric and/or small oligomeric species. To investigate the structural form of intracellular Tau, we washed the cells with 0.01% trypsin to digest any excess extracellular Tau before imaging by TCSPC. In contrast to the extracellular Tau fraction, we observed that both K18*-488 and hTau40*-488 proceeded to aggregate after uptake into cells. This is evident from a drop in the fluorescence lifetime from 3658 ± 16 ps before uptake to 3282 ± 72 ps within cells after 8-h incubation for K18*-488 and, correspondingly, from 3686 ± 16 ps to 3154 ± 56 ps for hTau40*-488 (Figs. 1*C, ii*, *iii*, *v*, and *vi*, and *D*, *Tau 8 h intracellular*, and 4, *medium blue bars*). These values are comparable with the fluorescence lifetime of K18*-488 aggregates formed *in vitro* in the presence of heparin (compare with Fig. 1*B*, *ii*, and *D*, K18* + *heparin*), hence suggesting that both intracellular and *in vitro* aggregates are in similar structural forms.

**FIGURE 2.**
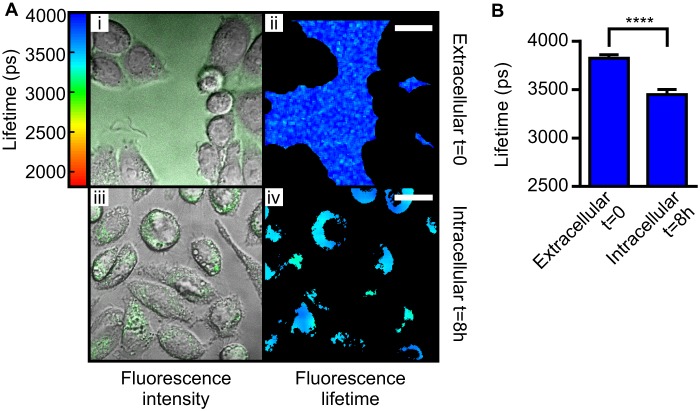
**Glycosaminoglycans are not involved in Tau uptake by cells.** *A*, PgsA-745 cells, which are deficient in glycosaminoglycans, were incubated with 1 μm 10% K18*-488. At t = 0, the fluorescence lifetime in the extracellular space was 3826 ± 36 ps, corresponding to soluble Tau. After 8 h, the cells were tryspin-washed, and the fluorescence lifetime of internalized K18*-488 was 3448 ± 54 ps, indicative of aggregation. *Scale bars* = 10 μm. *B*, *bar diagram* displaying the mean fluorescence lifetime values of the different samples measured. *Error bars* represent S.D. Unpaired Student's *t* test statistical analysis was performed. ****, *p* < 0.0001.

While investigating the mechanism of uptake, we noted that confocal images of SH-SY5Y cells exposed to exogenous Tau display a distinct punctate staining pattern suggesting that Tau localizes to vesicular structures (Fig. 1*C*, *ii* and *v*). Because glycosaminoglycans are present on the external side of the plasma membrane of cells and are known to act as membrane carriers, we investigated whether they are involved in the uptake of exogenous monomeric Tau. We thus incubated pgsA-745 cells, which are deficient in glycosaminoglycans (29), with 1 μm 10% K18*-488 (Fig. 2). Similar to the experiments described above using SH-SY5Y cells, K18*-488 is taken up by pgsA-745 cells, and within 8 h, the fluorescence lifetime was observed to drop to 3448 ± 54 ps, indicating that aggregation of K18* proceeded independently of the presence of glycosaminoglycans.

**FIGURE 3.**
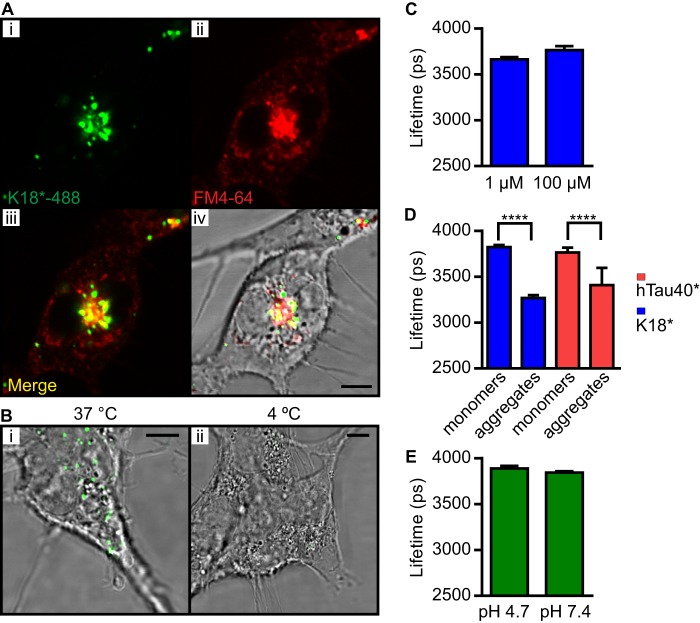
K18*-488 is taken up by endocytosis. *A*, SH-SY5Y cells were incubated with 1 μm 10% K18*-488 (*green*) for 24 h, and the lipid marker FM 4-64 (*red*) was added during the last 15 min of incubation. The cells were imaged by confocal microscopy. Colocalization between FM 4-64 and K18*-488 is displayed in *yellow. Scale bar* = 5 μm. *B*, SH-SY5Y cells were incubated with 1 μm 10% K18*-488 (*green*) either at 37 °C (*left panel*) or 4 °C (*right panel*). After 1 h, both dishes were trypsin-washed and imaged by confocal microscopy. The images display more internalized K18*-488 when the cells are incubated at 37 °C rather than at 4 °C. *Scale bars* = 5 μm. *C*, K18*-488 was incubated for 24 h at 100 μm in cell culture medium and compared with K18*-488 incubated at 1 μm in cell culture medium. The fluorescence lifetimes measured were 3765 ± 42 ps and 3664 ± 25 ps, respectively. *Error bars* represent S.D. *D*, K18*-488 was incubated at 1 μm (10% labeled) in cell culture medium at pH 4.7. hTau40* was incubated at 10 μm (1% labeled) in BES buffer at pH 4.7. The fluorescence lifetimes of soluble (3824 ± 22 ps) and aggregated K18*-488 (3268 ± 31 ps) and of soluble (3765 ± 53 ps) and aggregated hTau40*-488 (3409 ± 189 ps) indicate that low pH is sufficient to induce aggregation of both K18* and hTau40* *in vitro* in the absence of heparin. *Error bars* represent S.D. Unpaired Student's *t* test statistical analysis was performed. ****, *p* < 0.0001. *E*, Alexa Fluor 488 was dissolved in cell culture medium to 100 nm (pH 4.7 or 7.4), and the mean fluorescence lifetime was measured to be 3889 ps ± 28 ps and 3844 ± 14 ps, respectively. *Error bars* represent S.D.

To further investigate the uptake of Tau, we used FM 4-64, a vesicular marker (30), and studied its colocalization with K18*. Fig. 3*A* shows confocal microscopy images of K18*-488 (*green*) and FM 4-64 (*red*). A strong degree of colocalization (*yellow*) is observed, confirming that K18* localizes to vesicles. Furthermore, using SH-SY5Y cells, endocytosis was blocked by lowering the temperature to 4 °C for 1 h, which resulted in a pronounced reduction of K18* uptake. Fig. 3*B* is a comparison of uptake at 37 °C (Fig. 3*B*, *i*), displaying distinctly punctate staining inside the cell, with uptake at 4 °C (*ii*), when uptake is negligible.

Endocytotic vesicles can cause a local increase in the concentration of sequestered protein. We thus investigated whether high concentrations (100 μm) of K18*-488 can lead to protein aggregation *in vitro*. In Fig. 3*C*, we show that incubating 100 μm 1% K18*-488 for 24 h at 37 °C does not cause any fluorescence lifetime drop. Interestingly though, when we incubated 1 μm 10% K18*-488 or 10 μm 1% hTau40*-488 at pH 4.7, which represents the physiological environment inside endo- and lysosomes, we observed the formation of aggregates within 24 h and a corresponding decrease in fluorescence lifetime from 3824 ± 22 ps to 3268 ± 31 ps for K18*-488 and from 3765 ± 53 ps to 3409 ± 189 ps for hTau40*-488 (Fig. 3*D*), despite the absence of heparin. In contrast, a low pH value does not affect the fluorescence lifetime of the Alexa Fluor 488 fluorophore on its own in solution (*E*).

##### Exogenous Internalized Tau Seeds the Aggregation of Endogenous Tau in SH-SY5Y Cells

**FIGURE 4.**
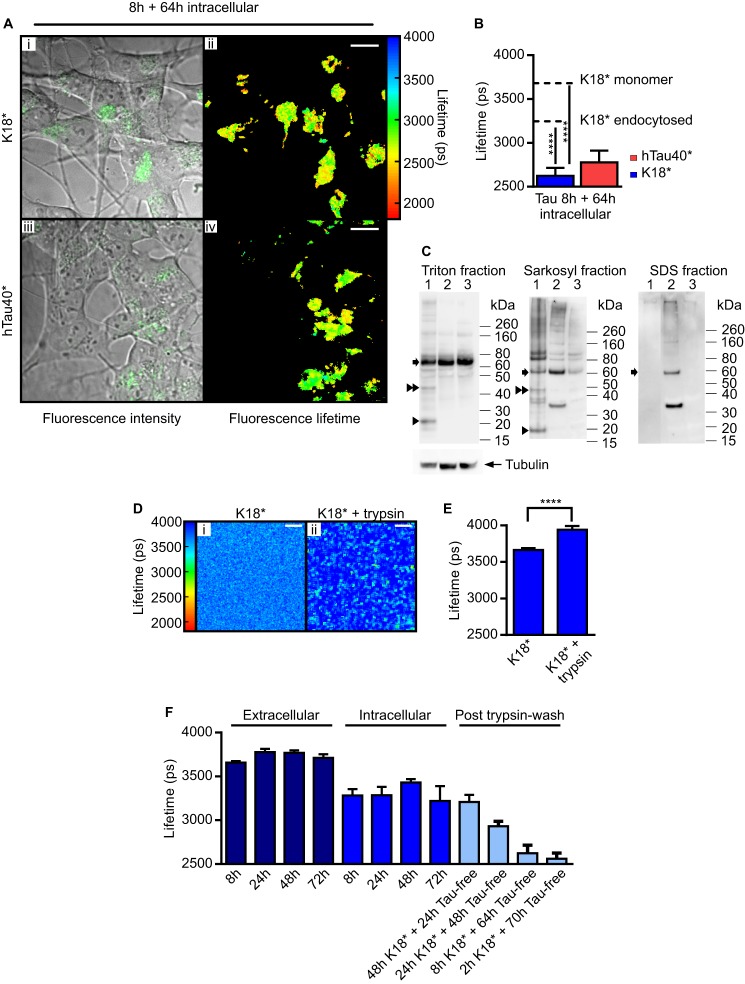
K18* seeds endogenous Tau aggregation. *A*, confocal and TCSPC images of SH-SY5Y cells incubated for 8 h with K18*-488 or hTau40*-488, respectively, trypsin-washed, and incubated in Tau-free medium for 64 h. *Scale bars* = 10 μm. *B*, corresponding *bar diagram* displaying the mean fluorescence lifetimes of K18*-488 (2624 ± 91 ps) and hTau40*-488 (2779 ± 132 ps), with *dashed lines* indicating fluorescence lifetimes of monomeric and endocytosed K18*. *Error bars* represent S.D. One-way analysis of variance was performed for K18* (F (2, 77) = 2080, *p* < 0.0001) and hTau40* (F (2, 56) = 352.5, *p* < 0.0001). ****, *p* < 0.0001. *C*, total Tau content was analyzed by Western blotting after sequential solubilization in Triton, sarkosyl, and SDS, respectively. *Lane 1*, cell lysates of cells incubated for 8 h with K18*. *Lane 2*, cell lysates of cells incubated for 8 h with K18*, trypsin-washed, and incubated for 64 h in Tau-free medium. *Lane 3*, cell lysates of age-matched control cells. *Single arrowheads* indicate monomeric K18*, *double arrowheads* point to K18* dimers, and *arrows* point to endogenous Tau. Tubulin was used as a loading control in the Triton-soluble fraction. *D* and *E*, 1 μm 10% K18*-488 was incubated for 24 h with 0.01% trypsin, and the fluorescence lifetime of K18*-488 was determined either prior to (*i*) or after (*ii*) trypsin incubation. The *bar diagram* represents the mean fluorescence lifetime of K18*-488 before (3664 ± 25 ps) or after trypsin incubation (3940 ± 50 ps). *Scale bars* = 10 μm. *Error bars* represent S.D. Unpaired Student's *t* test statistical analysis was performed. ****, *p* < 0.0001. *F*, SH-SY5Y cells were incubated with 1 μm 10% K18*-488, and the mean fluorescence lifetime of the fluorophore was recorded under the following conditions. *Navy blue*, extracellular space; *medium blue*, intracellular space; *light blue*: following uptake, trypsin-wash, and incubation in Tau-free medium. *Error bars* represent S.D.

Having established that K18* translocates into SH-SY5Y cells and leads to the formation of intracellular aggregates, we asked whether internalized K18* or hTau40* are capable of seeding the aggregation of endogenous Tau naturally present in SH-SY5Y cells. Fig. 4*A* shows fluorescence intensity (*left column*) and fluorescence lifetime (*right column*) images of 1 μm 10% K18*-488 (*i* and *ii*) and 1 μm 10% hTau40*-488 (*iii* and *iv*) after 8 h of incubation followed by trypsin wash and 64 h of incubation in Tau-free medium. Fluorescence lifetimes were observed to decrease to 2624 ± 91 ps for K18*-488 and to 2779 ± 132 ps for hTau40*-488. Depending on the time the cells were originally exposed to K18*-488 and then left in Tau-free medium, fluorescence lifetime decays were observed to decrease from 3208 ± 81 ps (Fig. 4*F*, 48 h K18* + *24 h Tau-free*) to 2561 ± 66 ps (2 h K18* + *70 h Tau-free*).

To investigate whether K18* is capable of inducing the aggregation of endogenous Tau, we sequentially extracted cell lysates of SH-SY5Y cells in 1% Triton, 1% sarkosyl, and 1% SDS. In Fig. 4*C*, we show that, after incubation with 1 μm K18* for 8 h, the cell lysates contain both monomeric (∼20-kDa) and dimeric (∼40-kDa) species of K18*, present both in the Triton- and sarkosyl-soluble fractions (*Triton fraction and Sarkosyl fraction*, *lanes 1*). Interestingly, cells incubated for 8 h with K18*, trypsin-washed, and then incubated for 64 h in Tau-free medium contained measurable amounts of endogenous Tau (∼60 kDa) in the SDS fractions of their lysates (Fig. 4*C*, *SDS fraction*, *lane 2*), whereas the lysates of control cells, not incubated with K18*, contained no endogenous Tau in the SDS fraction (*lane 3*). The presence of a band at 60 kDa in the SDS fraction is consistent with larger aggregates having formed containing endogenous Tau and, hence, supports the notion of K18* inducing the aggregation of endogenous Tau. Furthermore, in the lysates of cells that were incubated for 8 h with K18*, trypsin-washed, and then incubated for 64 h in Tau-free medium, neither K18* monomers nor dimers could be detected in the Triton-extracted fraction (Fig. 4*C*, *Triton fraction*, *lane 2*). The disappearance of these two species from the cells suggests that, after internalization, monomeric K18* is either degraded or forms higher-order oligomers.

To elucidate this and further investigate whether degradation of K18* is likely to take place inside cells, we modeled this process *in vitro* by observing the effect of trypsin on the fluorescence lifetime of K18*-488. We thus incubated 1 μm 10% K18*-488 with 0.01% trypsin for 24 h and found that trypsin causes an increase in fluorescence lifetime from 3664 ± 25 ps to 3940 ± 50 ps (Fig. 4, *D* and *E*). We attribute this observation to the digestion of Tau, to which the dye label is attached, so that the spectral properties of the fluorophore change and approach those of the free dye, which features a fluorescence lifetime of 4100 ps. However, during the cellular incubation process with K18*-488 over 8 h, followed by 64 h in Tau-free medium, a significant increase in the fluorescence lifetime was not observed (Fig. 4, *A* and *B*), suggesting that, upon internalization, the majority of K18* is not readily degraded but rather proceeds to aggregate further. We thus believe oligomerization to be the most likely process taking place inside the cells. This is further supported by the presence of a high molecular weight protein smear in the SDS fraction of cells incubated for 8 h with K18*, trypsin-washed, and then incubated for 64 h in Tau-free medium (Fig. 4*C*, *SDS fraction*, *lane 2*).

##### Exogenous K18* Propagates from Cell to Cell and Causes Further Aggregation of Endogenous Tau

**FIGURE 5.**
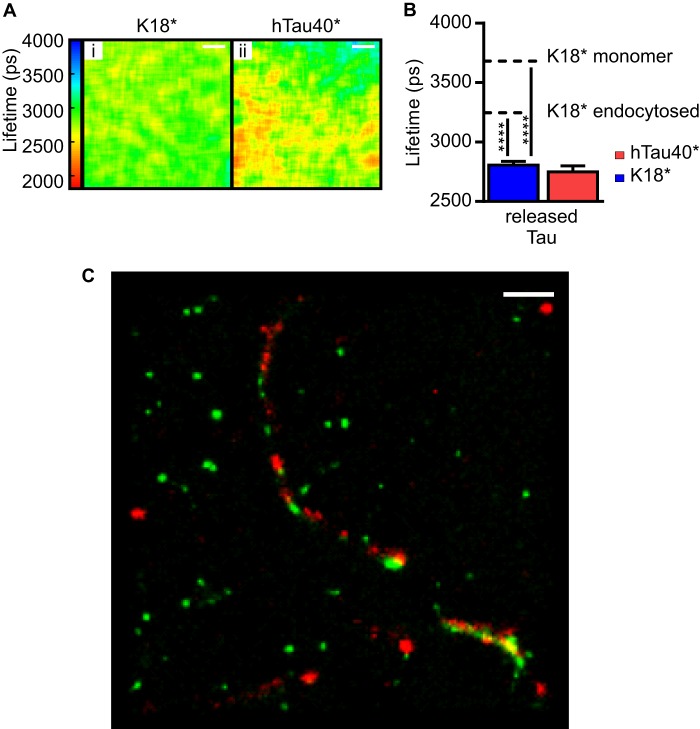
**Exogenously added and internalized Tau is released by cells.** *A*, SH-SY5Y cells were incubated with either K18*-488 or hTau40*-488 for 72 h, trypsin-washed, and incubated in Tau-free medium for 96 h prior to imaging. By analyzing the cell culture medium of these cells by TCSPC, the following fluorescence lifetimes for K18*-488 and hTau40*-488 were measured, respectively: 2807 ± 30 ps (*i*) and 2749 ± 49 ps (*ii*). *Scale bars* = 10 μm. *B*, the corresponding *bar diagram* represents the mean fluorescence lifetimes observed for released Tau. The *dotted lines* represent the mean fluorescence lifetime of monomeric and endocytosed K18*-488. *Error bars* represent S.D. One-way analysis of variance was performed for K18* (F (2, 62) = 1026, *p* < 0.0001) and hTau40* (F (2, 47) = 848.5, *p* < 0.0001). ****, *p* < 0.0001. *C*, the cell culture medium of cells treated with K18*-647 for 72 h, trypsin-washed, and incubated in Tau-free medium for 96 h was immunostained with the TAUY9 antibody prior to analysis by two-color *d*STORM. The image displays K18*-647 fibrils (*red*) that are intertwined with endogenous Tau (*green*). *Scale bar* = 1 μm.

**FIGURE 6.**
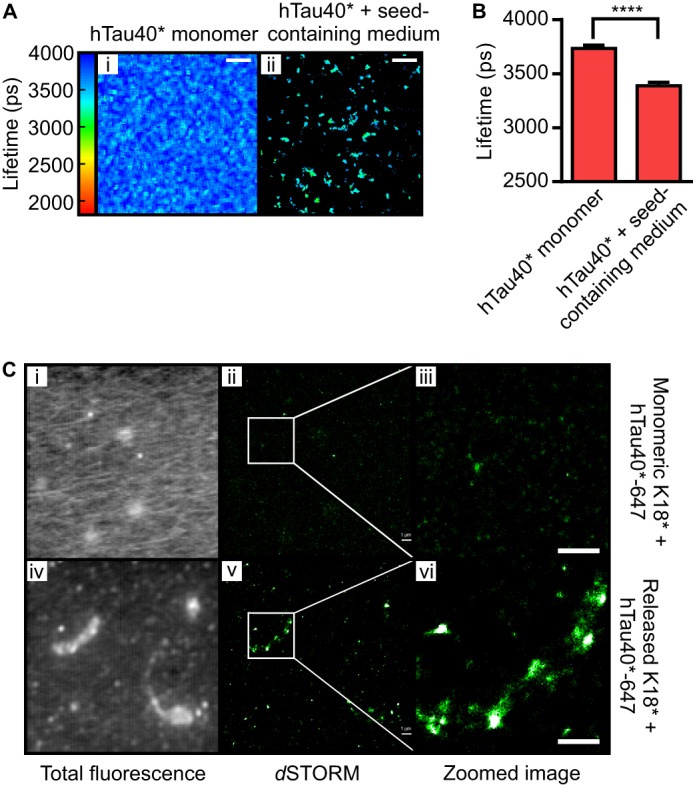
Released K18* seeds induce aggregation of full-length hTau40. *A*, culture medium containing released unlabeled K18* was incubated with 1 μm 10% hTau40*-488 for 48 h at 37 °C, and the mean fluorescence lifetime of hTau40*-488 was monitored before (*i*) and after (*ii*) incubation. *Scale bars* = 10 μm. *B*, *bar diagram* representing the mean fluorescence lifetime of hTau40*-488 prior (3735 ± 31 ps) and after incubation (3388 ± 33 ps) with culture medium containing released unlabeled K18*. *Error bars* represent S.D. Unpaired Student's *t* test statistical analysis was performed. ****, *p* < 0.0001. *C*, hTau40*-647 was incubated with soluble unlabeled K18* (*i*, *ii*, and *iii*) or with culture medium containing released K18* for 48 h at 37 °C (*iv*, *v*, and *vi*) prior to *d*STORM imaging. The fluorescence images of induced fibrils are displayed (*i* and *iv*). Color images show the corresponding (*ii* and *v*) and zoomed (*iii* and *vi*) superresolved images of the fibrils formed obtained by *d*STORM. hTau40*-647 incubated with medium containing released K18* forms fibrillar structures, whereas hTau40*-647 incubated with soluble K18* does not display any fibril formation. *Scale bars* = 1 μm.

Having shown that exogenously added monomeric K18* can aggregate when internalized by human neuroblastoma cells and that it subsequently induces aggregation of endogenous Tau, we asked whether aggregated Tau species can also exit the cells. We therefore incubated SH-SY5Y cells with 1 μm 10% K18*-488 or 1 μm 10% hTau40*-488 for 72 h, washed Tau with trypsin, and then incubated the cells for 96 h in Tau-free medium. We collected the extracellular medium and imaged it directly by TCSPC. Fig. 5, *A* and *B*, shows that the extracted extracellular medium displays low fluorescence lifetimes of 2807 ± 30 ps for K18*-488 and 2749 ± 49 ps for hTau40*-488, which indicate that the exocytosed material is in an aggregated form. We confirmed this result more directly with two-color *d*STORM. For this purpose, we repeated the above cell treatment with 1 μm 10% K18*-647, collected the extracellular medium, and made use of the TAUY9 primary antibody, which has been raised against amino acids 12–27 of full-length Tau and, therefore, recognizes an epitope outside of the K18 region. Crucially, the two-color assay revealed the presence of both K18*-647 and endogenous Tau in the released aggregates (Fig. 5*C*), clear evidence that the exogenous species can seed the formation of heterogeneous Tau aggregates. We investigated the reactivity of the species released via their propensity to induce aggregation of hTau40* *in vitro* using both fluorescence lifetime imaging and *d*STORM. We thus added 1 μm 10% hTau40*-488 or 1 μm 10% hTau40*-647 to the culture medium containing the unlabeled released species and performed TCSPC and *d*STORM after 48 h of incubation. We observed that hTau40*-488 fluorescence lifetimes decreased from 3735 ± 31 ps to 3388 ± 33 ps (Figs. 6, *A* and *B*) and that hTau40*-647-containing fibrils had formed upon incubation with Tau released from cells (*C*, *vi*), whereas fibril formation was not evident in a control experiment where hTau40*-647 was incubated with soluble K18* (*C*, *iii*).

**FIGURE 7.**
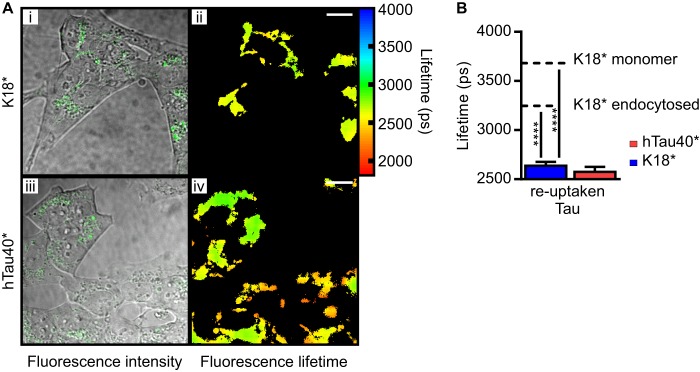
**Exogenously added and internalized Tau propagates from cell to cell.** *A*, the culture medium containing either released K18*-488 or released hTau40*-488 was transferred onto fresh cells and incubated for an additional 72 h. Re-uptaken K18*-488 displays a fluorescence lifetime of 2637 ± 40 ps (*ii*), and *i* displays the corresponding confocal image. Re-uptaken hTau40*-488 displays a fluorescence lifetime of 2575 ± 51 ps (*iv*), and *iii* displays the corresponding confocal image. *Scale bars* = 10 μm. *B*, corresponding *bar diagram* representing the mean fluorescence lifetimes observed for re-uptaken Tau. The *dotted lines* represent the mean fluorescence lifetime of monomeric and endocytosed K18*-488. *Error bars* represent S.D. One-way analysis of variance statistical analysis was performed for K18* (F (2, 72) = 2995, *p* < 0.0001) and hTau40* (F (2, 46) = 1120, *p* < 0.0001). ****, *p* < 0.0001.

Finally, we transferred the medium containing released Tau, either K18*-488 or hTau40*-488, onto fresh cells and incubated them for another 72 h before washing with trypsin and imaging by TCSPC. Confocal images (Fig. 7*A*, *i* and *iii*) display the appearance of labeled Tau fluorescence inside the cells, thus confirming the reuptake of Tau seeds by healthy cells. Corresponding fluorescence lifetimes were measured to be 2637 ± 40 ps inside the cells for K18*-488 and 2575 ± 51 ps for hTau40*-488 (Fig. 7*A*, *ii* and *iv*, and *B*), indicating that further aggregation has occurred.

## DISCUSSION

**FIGURE 8.**
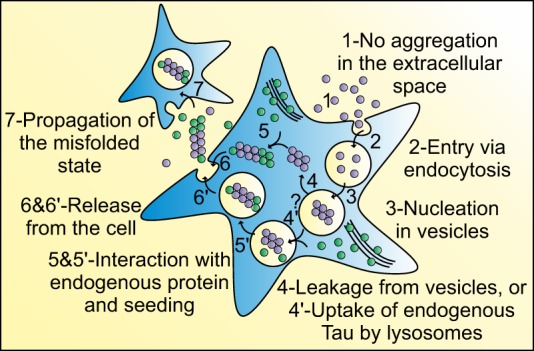
**Monomeric Tau initiates the misfolding cycle.** Shown is a proposed model of the spread of Tau pathology. Intracellular monomeric Tau is released into the extracellular space through processes involving neuronal death or exocytosis, which is supported by elevated levels of Tau measured in the cerebrospinal fluid of AD patients. Monomeric Tau (*1*) can now directly be taken up by surrounding neurons through endocytosis (*2*). The intravesicular environment, such as present in endo- or lysosomes, then promotes the nucleation of endocytosed Tau (*3*). Our model does not currently address the question whether Tau leaves the intravesicular compartment or not. Thus, exogenous Tau can either be released into the cytoplasm (*4*), where it can seed aggregation of endogenous healthy Tau (*5*) or encounter endogenous Tau targeted for intravesicular degradation (*4*′) and seed the latter (*5*′). Heterogeneous aggregates are consequently released into the extracellular space either through not yet defined pathways such as exosomal release or simply by cell death (*6*) or by lysosomal exocytosis (*6*′). Thus, the propagation of misfolded Tau can now proceed as suggested previously (*7*).

The ability of Tau to eventually form neurofibrillary tangles is central to the onset and progression of neurodegeneration in AD. The challenges involved in directly monitoring the dynamics of protein conformations in live specimens have, however, made it difficult to define the molecular events that initiate Tau aggregation and the proliferation of ensuing aggregates *in vivo*. This holds true particularly for the transfer of Tau from one cell to the next in an aggregated and potentially toxic conformation, which may be the causative event behind the stereotypic spreading observed in AD. Here, we used a combination of novel fluorescence-based microscopy techniques to overcome these limitations, enabling the real-time study of Tau aggregation dynamics and its trafficking between and into cells, and our data provide a new model on the spread of Tau pathology (Fig. 8).

### 

#### 

##### The Fluorescence Lifetime of Labeled Tau Variants Offers Information on Their Structural State

We have shown previously that the fluorescence lifetime of a dye attached to an aggregation-prone protein is sensitive to the structural transition from random coil to cross-β sheet fibril and that, thus, the fluorescence lifetime is reduced in an aggregation-dependent manner (19), permitting us to distinguish between monomeric, oligomeric, and fibrillar structures. In this study, we apply the same principle and show, through *in vitro* characterization of the aggregation of Alexa Fluor 488-labeled hTau40* and K18*, that the formation of β sheet-rich fibrillar species is accompanied by reductions in the Alexa Fluor 488 fluorescence lifetime and demonstrate that this effect can be measured *via* TCSPC. The fluorescence lifetime of Alexa Fluor 488 dye labels attached to Tau monomers provides a sensitive and robust readout of aggregation progression, with the advantage of insensitivity to environmental changes such as pH or concentration quenching. The probe is thus particularly well suited for monitoring pathologic aggregation *in vivo*, offering structural information that is not available via intensity-based fluorescence measurements (31).

##### Monomeric Forms of K18* and hTau40* Are Taken Up by Neuron-like Cells

Using fluorescence lifetime imaging and Western blotting, we confirmed that both K18* and hTau40* are taken up in monomeric form and that no measurable oligomerization is necessary prior to entry. Our findings for K18* are consistent with those by Frost *et al.* (11), who found that K18 monomers are taken up by neural C17.2 cells. For the full-length hTau40*, there are no corresponding data in the literature. In fact, a recent study by Wu *et al.* (16) indicated that, at least in primary mouse hippocampal and cortical neurons, the monomeric form of exogenously added hTau40 is not internalized. The discrepancy between the study of Wu and our study may stem from the different sensitivity of the systems used to measure the amount of monomeric protein in cells, for which TCSPC, used here, is particularly well suited because of its single photon detection capability.

##### Endosomes Are Conducive to the Nucleation of Monomeric Tau into Aggregates

We show, furthermore, that monomeric K18* and hTau40* are taken up via pathways that are partially blocked by low temperature, which is consistent with endocytotic uptake. When in endosomes, Tau is subjected to a number of physicochemical factors that are likely to exert an influence on ensuing aggregation reactions. In particular, endosomal pH is reported to vary between 5.0 and 6.0 and lysosomal pH between 4.6 and 5.0 (32). Aggregation may be favored by the low pH in these compartments, as supported by our results *in vitro* and other factors, such as molecular crowding or the presence of acidic endosomal proteins, suggestive of endocytosis as a crucial mechanism facilitating Tau aggregation and propagation. We have shown previously that a low pH value assists in the aggregation of Tau in the absence of heparin (33), and others have reported that the accumulation of Tau aggregates in endosomes and lysosomes has been observed (11, 12, 16). However, those reports all emphasize the importance of endocytosis as the mechanism for the internalization of preformed Tau fibrils, whereas here, we establish a clear link between the process of endocytosis of monomeric Tau, its nucleation, and the formation of infectious aggregate seeds. This finding could be the crucial first step in the initiation and propagation of the disease. The consequences are far-reaching because the mere presence of soluble Tau in the extracellular space could lead to aggregation upon endocytosis and possible formation of toxic species. This would be consistent with clinical observations of increased levels of neurofibrillary tangles found in patients after traumatic brain injury (34) because our study indicates that the release of small amounts of soluble Tau from dying neurons into the cerebrospinal fluid may be sufficient to trigger Tau pathology. Whether or not extracellular monomeric Tau is directly correlated with increased neurotoxicity remains to be determined. However, our data suggest that increased levels of exogenous Tau may cause a greater pathological risk to patients than hitherto suspected. Thus, the clearance of Tau from the cerebrospinal fluid or blocking of endocytosis pathways may provide viable avenues for future therapeutic intervention.

##### Internalized and Subsequently Aggregated Tau Proteins Act as Seeds for the Aggregation of Endogenous Tau

The interaction of endocytosed Tau with cytosolic Tau has been shown previously, but only in the case of cells overexpressing Tau (11, 12, 15). Furthermore, in the study by Guo and Lee (12), the authors internalized Tau with the help of BioPORTER®, a cationic lipid designed to assist the delivery of proteins into cells and, thus, targeting the protein directly to the cytoplasm. However, we have shown that exogenously added and subsequently internalized and aggregated, Tau is capable of seeding the aggregation of endogenous Tau in SH-SY5Y cells. We verified, using Western blotting, that endogenous Tau is present in the sarkosyl-insoluble/SDS-soluble aggregate fraction. Two scenarios are likely to occur. First, there is leakage of endosomal and lysosomal content into the cytosol. Indeed, this has been observed in the related field of Aβ_1–42_ internalization (35, 36), and it is conceivable that Tau, which has been shown, in a recent study, to disrupt synthetic lipid bilayers (37), may also cause leakage of endo- or lysosomal content. Second, coaggregation occurs in the late endo-lysosomal compartment. In cells, newly synthesized Tau is degraded within 16 h.^4^ Thus, it is likely for nucleated exogenous Tau to be exposed to endogenous Tau that has been taken up by lysosomes (38) within the time frame of our experimental setup (72 h). This scenario, if confirmed, would provide a model of how coaggregated exogenous and endogenous Tau can exit the cells via lysosomal exocytosis, similar to what was observed previously for the related case of α-synuclein trafficking (39).

##### Intracellular Coaggregates of Tau Can Exit Infected Cells

We furthermore observed, by two-color superresolution imaging, that aggregates containing mixtures of exogenous and endogenous Tau are released by cells. Using antibodies specific for the endogenous full-length protein and labeled K18*, two-color *d*STORM imaging verified that exogenously added Tau can seed the aggregation of endogenous Tau and that the resulting coaggregates are subsequently released into the extracellular space. Release of Tau by cells has been documented previously, but the mechanism of release is still a matter of debate. Indeed, recent studies report either the association of Tau with exosomes (17, 40) or the release of Tau monomers through an active mechanism independent of exosomes (18, 41, 42). These diverging results may be due to a sensitivity of Tau association with exosomes on intracellular concentration of the protein, highlighting potential problems with the use of protein overexpression systems.

In conclusion, we have demonstrated that hTau40*-488 and K18*-488 form aggregates with characteristic fluorescence lifetime signatures and morphological features consistent with the formation of fibrils in endosomal compartments following the uptake of Tau. Taken together, our data support the notion that a short exposure to monomeric extracellular Tau leads to the infection of healthy cells and initiates the nucleation of aggregate seeds that incorporate and rapidly progress the aggregation of endogenous Tau. The resulting coaggregates are furthermore seen to be released into the extracellular medium and are capable of infecting other cells, which has also been shown by other authors (1, 12, 15, 16). The data suggest that the aggregation of Tau *in vivo* is primarily controlled by its sensitivity to the endosomal environment and, thus, emphasizes the role of endocytosis in the spread of pathological species.

## Footnotes

4 U. Krüger and E.-M. Mandelkow, unpublished data.

## Footnotes

3 The abbreviations used are:

AD: Alzheimer disease
Aβ: amyloid β
BES: N,N-bis(2-hydroxyethyl)taurine
MEM: minimal essential medium
TCSPC: time-correlated single photon counting
*d*STORM: direct stochastic optical reconstruction microscopy
ps: picosecond.
